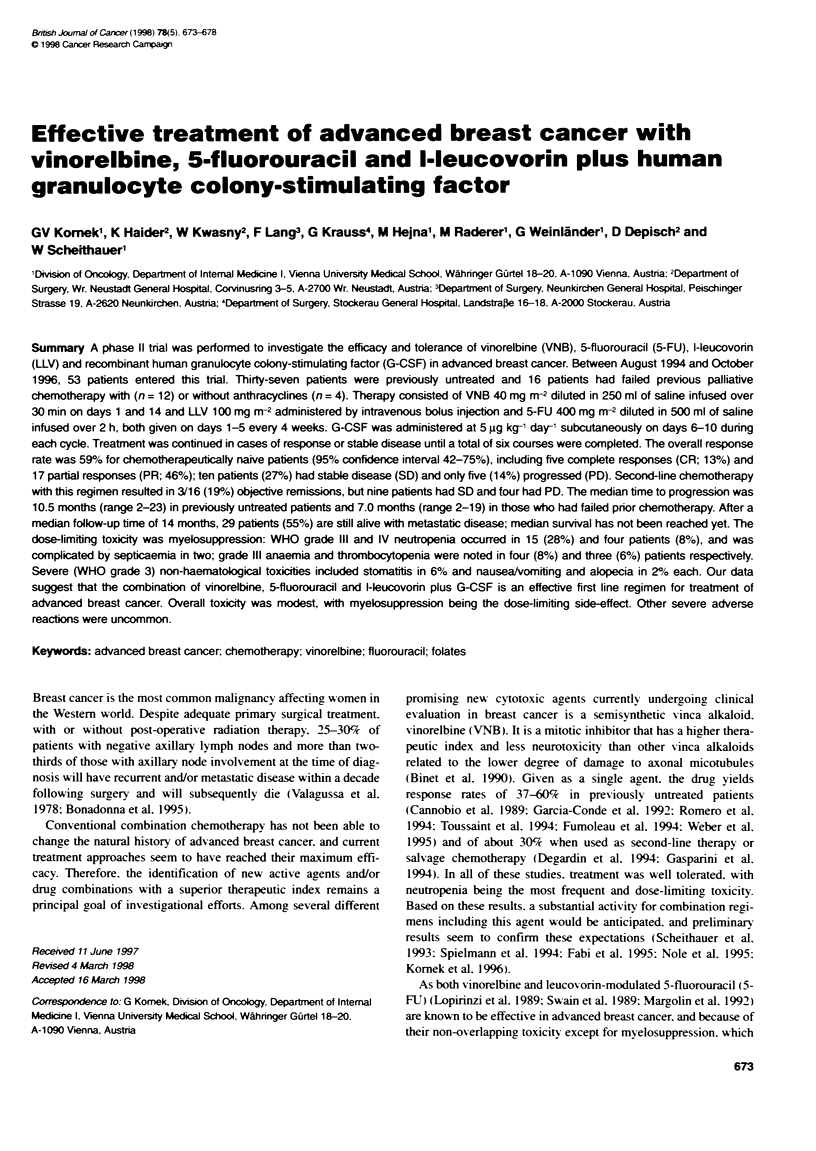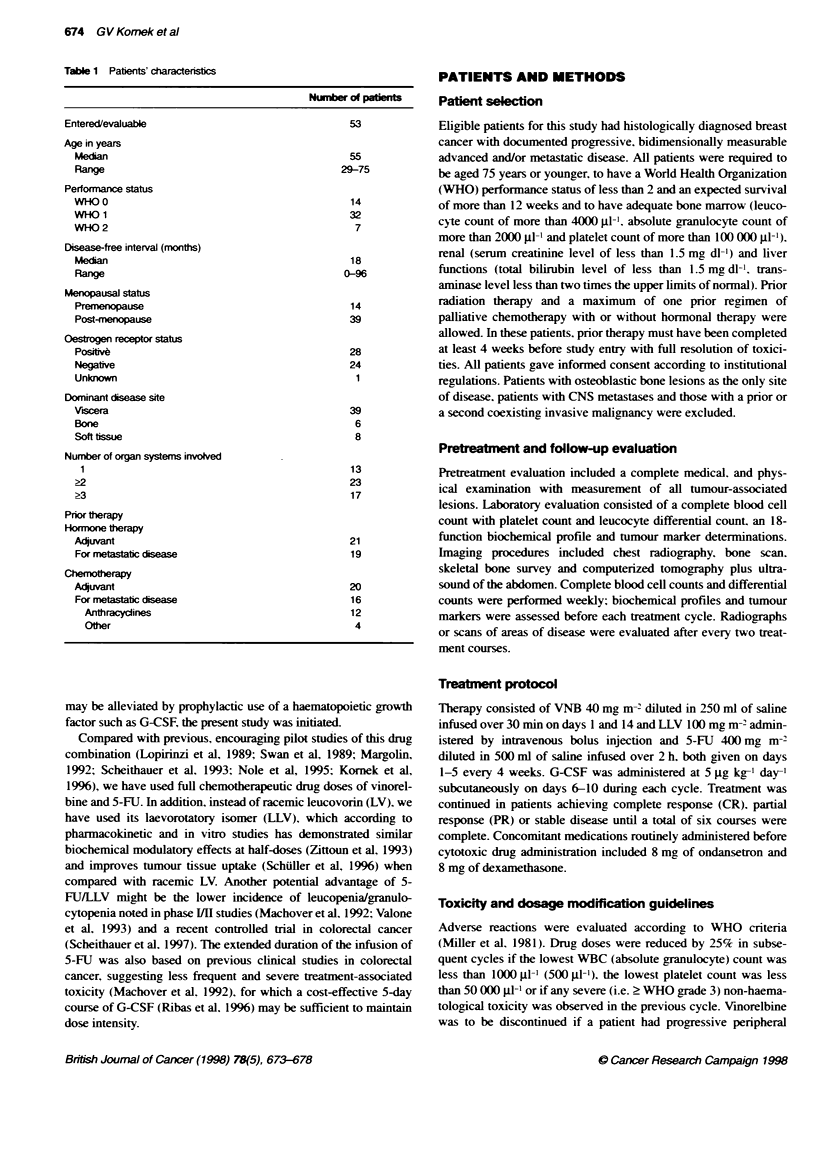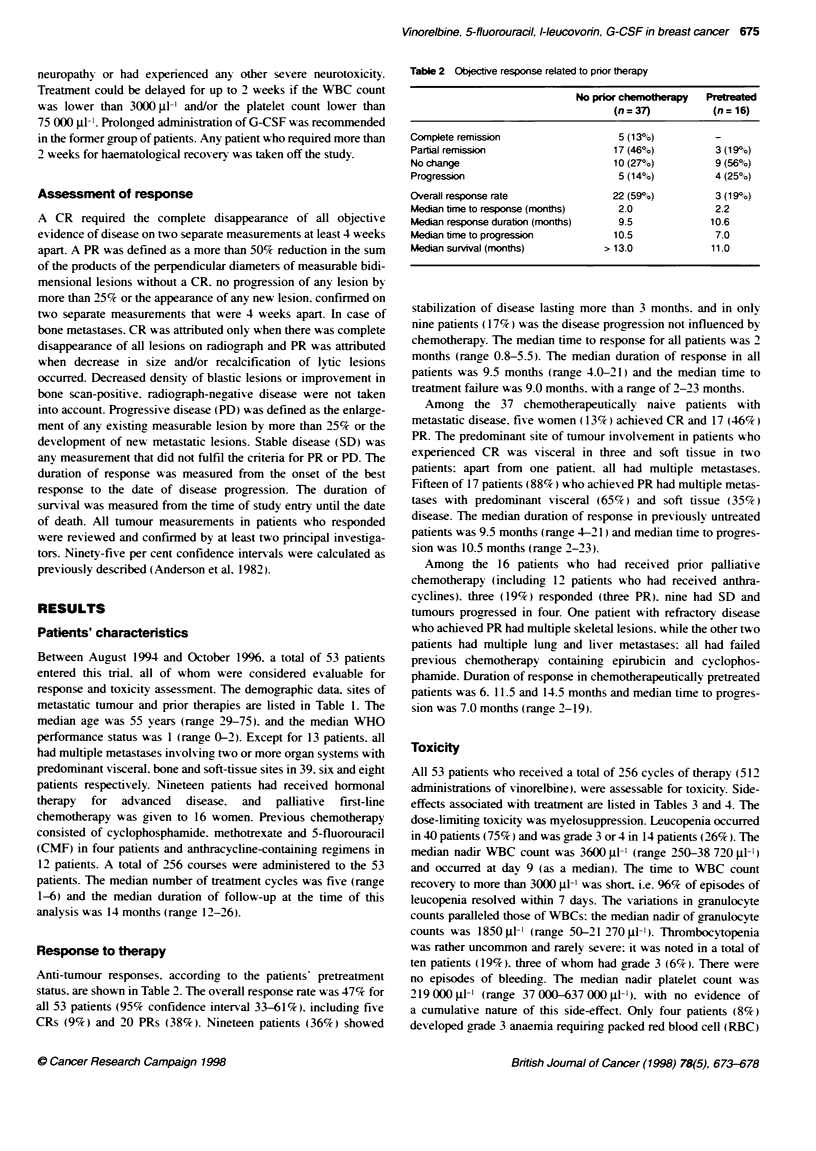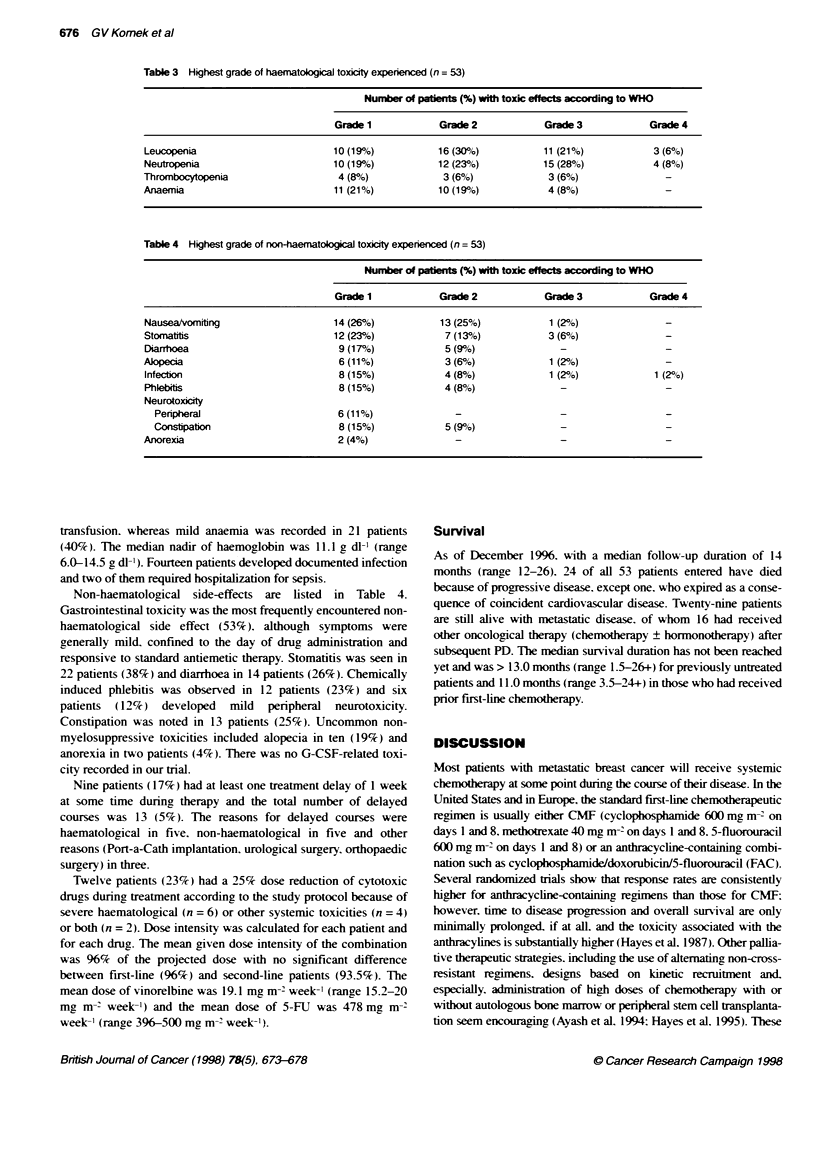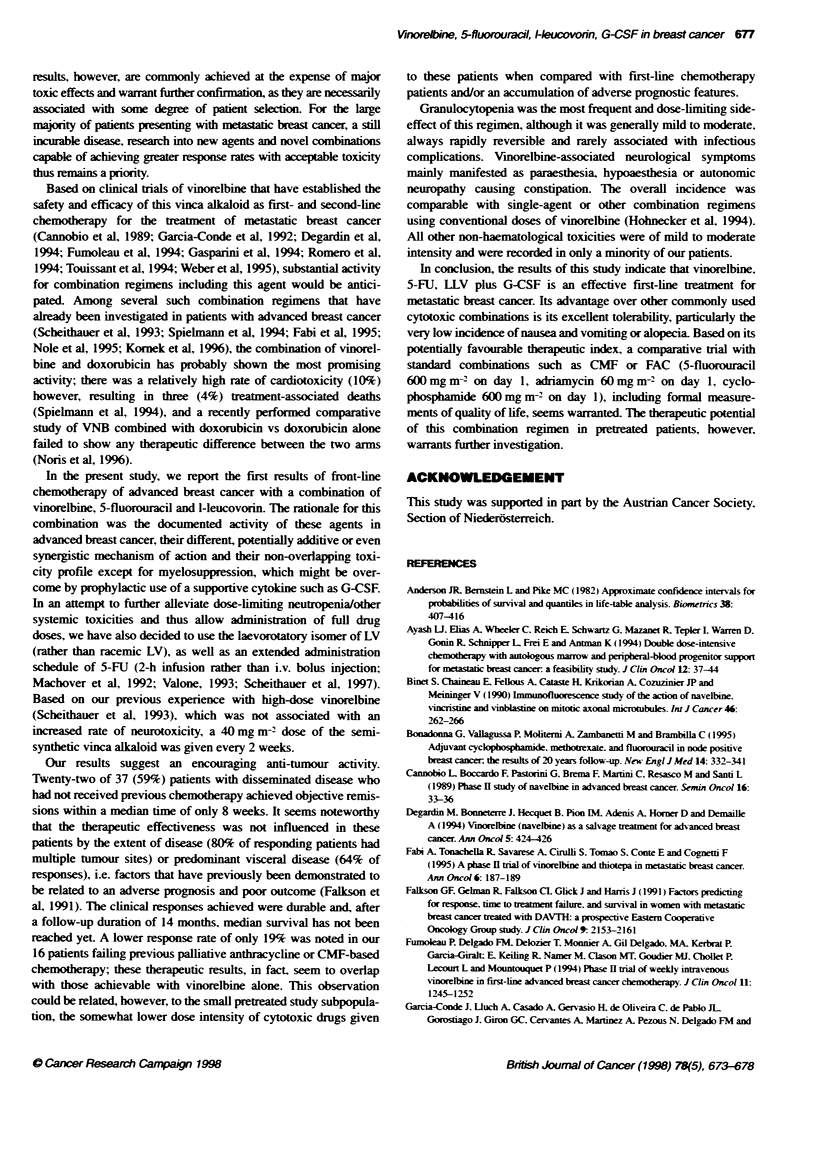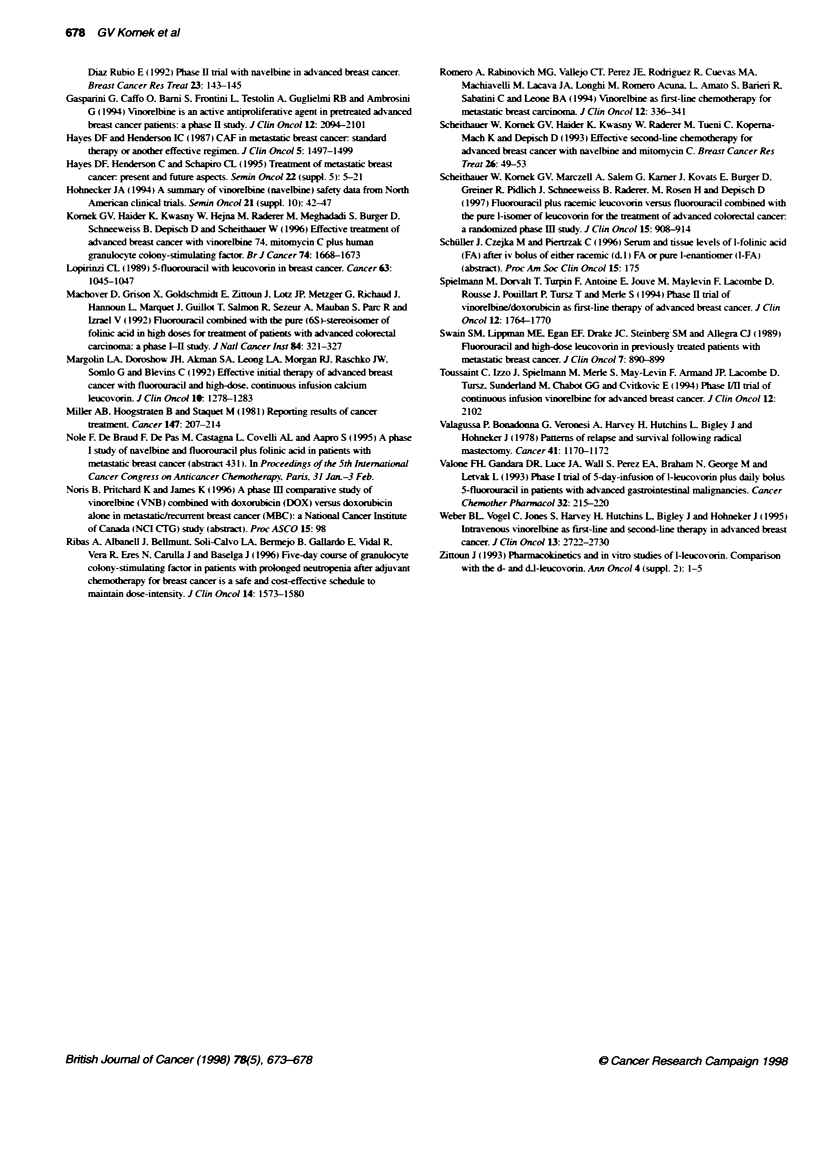# Effective treatment of advanced breast cancer with vinorelbine, 5-fluorouracil and l-leucovorin plus human granulocyte colony-stimulating factor.

**DOI:** 10.1038/bjc.1998.558

**Published:** 1998-09

**Authors:** G. V. Kornek, K. Haider, W. Kwasny, F. Lang, G. Krauss, M. Hejna, M. Raderer, G. Weinländer, D. Depisch, W. Scheithauer

**Affiliations:** Department of Internal Medicine I, Vienna University Medical School, Austria.

## Abstract

A phase II trial was performed to investigate the efficacy and tolerance of vinorelbine (VNB), 5-fluorouracil (5-FU), l-leucovorin (LLV) and recombinant human granulocyte colony-stimulating factor (G-CSF) in advanced breast cancer. Between August 1994 and October 1996, 53 patients entered this trial. Thirty-seven patients were previously untreated and 16 patients had failed previous palliative chemotherapy with (n = 12) or without anthracyclines (n = 4). Therapy consisted of VNB 40 mg m(-2) diluted in 250 ml of saline infused over 30 min on days 1 and 14 and LLV 100 mg m(-2) administered by intravenous bolus injection and 5-FU 400 mg m(-2) diluted in 500 ml of saline infused over 2 h, both given on days 1-5 every 4 weeks. G-CSF was administered at 5 microg kg(-1) day(-1) subcutaneously on days 6-10 during each cycle. Treatment was continued in cases of response or stable disease until a total of six courses were completed. The overall response rate was 59% for chemotherapeutically naive patients (95% confidence interval 42-75%), including five complete responses (CR; 13%) and 17 partial responses (PR; 46%); ten patients (27%) had stable disease (SD) and only five (14%) progressed (PD). Second-line chemotherapy with this regimen resulted in 3/16 (19%) objective remissions, but nine patients had SD and four had PD. The median time to progression was 10.5 months (range 2-23) in previously untreated patients and 7.0 months (range 2-19) in those who had failed prior chemotherapy. After a median follow-up time of 14 months, 29 patients (55%) are still alive with metastatic disease; median survival has not been reached yet. The dose-limiting toxicity was myelosuppression: WHO grade III and IV neutropenia occurred in 15 (28%) and four patients (8%), and was complicated by septicaemia in two; grade III anaemia and thrombocytopenia were noted in four (8%) and three (6%) patients respectively. Severe (WHO grade 3) non-haematological toxicities included stomatitis in 6% and nausea/vomiting and alopecia in 2% each. Our data suggest that the combination of vinorelbine, 5-fluorouracil and l-leucovorin plus G-CSF is an effective first line regimen for treatment of advanced breast cancer. Overall toxicity was modest, with myelosuppression being the dose-limiting side-effect. Other severe adverse reactions were uncommon.


British Journal of Cancer (1998) 78(5), 673–678
© 1998 Cancer Research Campaign

### Effective treatment of advanced breast cancer with vinorelbine, 5-fluorouracil and l-leucovorin plus human granulocyte colony-stimulating factor

GV Kornek[1], K Haider[2], W Kwasny[2], F Lang[3], G Krauss[4], M Hejna[1], M Raderer[1], G Weinländer[1], D Depisch[2] and W Scheithauer[1]

[1]Division of Oncology, Department of Internal Medicine I, Vienna University Medical School, Währinger Gürtel 18–20, A-1090 Vienna, Austria; [2]Department of Surgery, Wr. Neustadt General Hospital, Corvinusring 3–5, A-2700 Wr. Neustadt, Austria; [3]Department of Surgery, Neunkirchen General Hospital, Peischinger Strasse 19, A-2620 Neunkirchen, Austria; [4]Department of Surgery, Stockerau General Hospital, Landstraße 16–18, A-2000 Stockerau, Austria

**Summary** A phase II trial was performed to investigate the efficacy and tolerance of vinorelbine (VNB), 5-fluorouracil (5-FU), l-leucovorin (LLV) and recombinant human granulocyte colony-stimulating factor (G-CSF) in advanced breast cancer. Between August 1994 and October 1996, 53 patients entered this trial. Thirty-seven patients were previously untreated and 16 patients had failed previous palliative chemotherapy with ($n$ = 12) or without anthracyclines ($n$ = 4). Therapy consisted of VNB 40 mg $m^{-2}$ diluted in 250 ml of saline infused over 30 min on days 1 and 14 and LLV 100 mg $m^{-2}$ administered by intravenous bolus injection and 5-FU 400 mg $m^{-2}$ diluted in 500 ml of saline infused over 2 h, both given on days 1–5 every 4 weeks. G-CSF was administered at 5 μg $kg^{-1}$ $day^{-1}$ subcutaneously on days 6–10 during each cycle. Treatment was continued in cases of response or stable disease until a total of six courses were completed. The overall response rate was 59% for chemotherapeutically naive patients (95% confidence interval 42–75%), including five complete responses (CR; 13%) and 17 partial responses (PR; 46%); ten patients (27%) had stable disease (SD) and only five (14%) progressed (PD). Second-line chemotherapy with this regimen resulted in 3/16 (19%) objective remissions, but nine patients had SD and four had PD. The median time to progression was 10.5 months (range 2–23) in previously untreated patients and 7.0 months (range 2–19) in those who had failed prior chemotherapy. After a median follow-up time of 14 months, 29 patients (55%) are still alive with metastatic disease; median survival has not been reached yet. The dose-limiting toxicity was myelosuppression: WHO grade III and IV neutropenia occurred in 15 (28%) and four patients (8%), and was complicated by septicaemia in two; grade III anaemia and thrombocytopenia were noted in four (8%) and three (6%) patients respectively. Severe (WHO grade 3) non-haematological toxicities included stomatitis in 6% and nausea/vomiting and alopecia in 2% each. Our data suggest that the combination of vinorelbine, 5-fluorouracil and l-leucovorin plus G-CSF is an effective first line regimen for treatment of advanced breast cancer. Overall toxicity was modest, with myelosuppression being the dose-limiting side-effect. Other severe adverse reactions were uncommon.

**Keywords:** advanced breast cancer; chemotherapy; vinorelbine; fluorouracil; folates

Breast cancer is the most common malignancy affecting women in the Western world. Despite adequate primary surgical treatment, with or without post-operative radiation therapy, 25–30% of patients with negative axillary lymph nodes and more than two-thirds of those with axillary node involvement at the time of diagnosis will have recurrent and/or metastatic disease within a decade following surgery and will subsequently die (Valagussa et al, 1978; Bonadonna et al, 1995).

Conventional combination chemotherapy has not been able to change the natural history of advanced breast cancer, and current treatment approaches seem to have reached their maximum efficacy. Therefore, the identification of new active agents and/or drug combinations with a superior therapeutic index remains a principal goal of investigational efforts. Among several different promising new cytotoxic agents currently undergoing clinical evaluation in breast cancer is a semisynthetic vinca alkaloid, vinorelbine (VNB). It is a mitotic inhibitor that has a higher therapeutic index and less neurotoxicity than other vinca alkaloids related to the lower degree of damage to axonal micotubules (Binet et al, 1990). Given as a single agent, the drug yields response rates of 37–60% in previously untreated patients (Cannobio et al, 1989; Garcia-Conde et al, 1992; Romero et al, 1994; Toussaint et al, 1994; Fumoleau et al, 1994; Weber et al, 1995) and of about 30% when used as second-line therapy or salvage chemotherapy (Degardin et al, 1994; Gasparini et al, 1994). In all of these studies, treatment was well tolerated, with neutropenia being the most frequent and dose-limiting toxicity. Based on these results, a substantial activity for combination regimens including this agent would be anticipated, and preliminary results seem to confirm these expectations (Scheithauer et al, 1993; Spielmann et al, 1994; Fabi et al, 1995; Nole et al, 1995; Kornek et al, 1996).

As both vinorelbine and leucovorin-modulated 5-fluorouracil (5-FU) (Lopirinzi et al, 1989; Swain et al, 1989; Margolin et al, 1992) are known to be effective in advanced breast cancer, and because of their non-overlapping toxicity except for myelosuppression, which

*Received 11 June 1997*
*Revised 4 March 1998*
*Accepted 16 March 1998*

*Correspondence to:* G Kornek, Division of Oncology, Department of Internal Medicine I, Vienna University Medical School, Währinger Gürtel 18–20, A-1090 Vienna, Austria

Table 1 Patients' characteristics

| | Number of patients |
|---|---|
| Entered/evaluable | 53 |
| Age in years | |
| Median | 55 |
| Range | 29–75 |
| Performance status | |
| WHO 0 | 14 |
| WHO 1 | 32 |
| WHO 2 | 7 |
| Disease-free interval (months) | |
| Median | 18 |
| Range | 0–96 |
| Menopausal status | |
| Premenopause | 14 |
| Post-menopause | 39 |
| Oestrogen receptor status | |
| Positive | 28 |
| Negative | 24 |
| Unknown | 1 |
| Dominant disease site | |
| Viscera | 39 |
| Bone | 6 |
| Soft tissue | 8 |
| Number of organ systems involved | |
| 1 | 13 |
| ≥2 | 23 |
| ≥3 | 17 |
| Prior therapy | |
| Hormone therapy | |
| Adjuvant | 21 |
| For metastatic disease | 19 |
| Chemotherapy | |
| Adjuvant | 20 |
| For metastatic disease | 16 |
| Anthracyclines | 12 |
| Other | 4 |

may be alleviated by prophylactic use of a haematopoietic growth factor such as G-CSF, the present study was initiated.

Compared with previous, encouraging pilot studies of this drug combination (Lopirinzi et al, 1989; Swan et al, 1989; Margolin, 1992; Scheithauer et al, 1993; Nole et al, 1995; Kornek et al, 1996), we have used full chemotherapeutic drug doses of vinorelbine and 5-FU. In addition, instead of racemic leucovorin (LV), we have used its laevorotatory isomer (LLV), which according to pharmacokinetic and in vitro studies has demonstrated similar biochemical modulatory effects at half-doses (Zittoun et al, 1993) and improves tumour tissue uptake (Schüller et al, 1996) when compared with racemic LV. Another potential advantage of 5-FU/LLV might be the lower incidence of leucopenia/granulocytopenia noted in phase I/II studies (Machover et al, 1992; Valone et al, 1993) and a recent controlled trial in colorectal cancer (Scheithauer et al, 1997). The extended duration of the infusion of 5-FU was also based on previous clinical studies in colorectal cancer, suggesting less frequent and severe treatment-associated toxicity (Machover et al, 1992), for which a cost-effective 5-day course of G-CSF (Ribas et al, 1996) may be sufficient to maintain dose intensity.

### PATIENTS AND METHODS

### Patient selection

Eligible patients for this study had histologically diagnosed breast cancer with documented progressive, bidimensionally measurable advanced and/or metastatic disease. All patients were required to be aged 75 years or younger, to have a World Health Organization (WHO) performance status of less than 2 and an expected survival of more than 12 weeks and to have adequate bone marrow (leucocyte count of more than 4000 $\mu l^{-1}$, absolute granulocyte count of more than 2000 $\mu l^{-1}$ and platelet count of more than 100 000 $\mu l^{-1}$), renal (serum creatinine level of less than 1.5 mg $dl^{-1}$) and liver functions (total bilirubin level of less than 1.5 mg $dl^{-1}$, transaminase level less than two times the upper limits of normal). Prior radiation therapy and a maximum of one prior regimen of palliative chemotherapy with or without hormonal therapy were allowed. In these patients, prior therapy must have been completed at least 4 weeks before study entry with full resolution of toxicities. All patients gave informed consent according to institutional regulations. Patients with osteoblastic bone lesions as the only site of disease, patients with CNS metastases and those with a prior or a second coexisting invasive malignancy were excluded.

### Pretreatment and follow-up evaluation

Pretreatment evaluation included a complete medical, and physical examination with measurement of all tumour-associated lesions. Laboratory evaluation consisted of a complete blood cell count with platelet count and leucocyte differential count, an 18-function biochemical profile and tumour marker determinations. Imaging procedures included chest radiography, bone scan, skeletal bone survey and computerized tomography plus ultrasound of the abdomen. Complete blood cell counts and differential counts were performed weekly; biochemical profiles and tumour markers were assessed before each treatment cycle. Radiographs or scans of areas of disease were evaluated after every two treatment courses.

### Treatment protocol

Therapy consisted of VNB 40 mg $m^{-2}$ diluted in 250 ml of saline infused over 30 min on days 1 and 14 and LLV 100 mg $m^{-2}$ administered by intravenous bolus injection and 5-FU 400 mg $m^{-2}$ diluted in 500 ml of saline infused over 2 h, both given on days 1–5 every 4 weeks. G-CSF was administered at 5 $\mu g\ kg^{-1}\ day^{-1}$ subcutaneously on days 6–10 during each cycle. Treatment was continued in patients achieving complete response (CR), partial response (PR) or stable disease until a total of six courses were complete. Concomitant medications routinely administered before cytotoxic drug administration included 8 mg of ondansetron and 8 mg of dexamethasone.

### Toxicity and dosage modification guidelines

Adverse reactions were evaluated according to WHO criteria (Miller et al, 1981). Drug doses were reduced by 25% in subsequent cycles if the lowest WBC (absolute granulocyte) count was less than 1000 $\mu l^{-1}$ (500 $\mu l^{-1}$), the lowest platelet count was less than 50 000 $\mu l^{-1}$ or if any severe (i.e. ≥ WHO grade 3) non-haematological toxicity was observed in the previous cycle. Vinorelbine was to be discontinued if a patient had progressive peripheral

© Cancer Research Campaign 1998

neuropathy or had experienced any other severe neurotoxicity. Treatment could be delayed for up to 2 weeks if the WBC count was lower than 3000 μl$^{-1}$ and/or the platelet count lower than 75 000 μl$^{-1}$. Prolonged administration of G-CSF was recommended in the former group of patients. Any patient who required more than 2 weeks for haematological recovery was taken off the study.

### Assessment of response

A CR required the complete disappearance of all objective evidence of disease on two separate measurements at least 4 weeks apart. A PR was defined as a more than 50% reduction in the sum of the products of the perpendicular diameters of measurable bidimensional lesions without a CR, no progression of any lesion by more than 25% or the appearance of any new lesion, confirmed on two separate measurements that were 4 weeks apart. In case of bone metastases, CR was attributed only when there was complete disappearance of all lesions on radiograph and PR was attributed when decrease in size and/or recalcification of lytic lesions occurred. Decreased density of blastic lesions or improvement in bone scan-positive, radiograph-negative disease were not taken into account. Progressive disease (PD) was defined as the enlargement of any existing measurable lesion by more than 25% or the development of new metastatic lesions. Stable disease (SD) was any measurement that did not fulfil the criteria for PR or PD. The duration of response was measured from the onset of the best response to the date of disease progression. The duration of survival was measured from the time of study entry until the date of death. All tumour measurements in patients who responded were reviewed and confirmed by at least two principal investigators. Ninety-five per cent confidence intervals were calculated as previously described (Anderson et al, 1982).

### RESULTS

### Patients' characteristics

Between August 1994 and October 1996, a total of 53 patients entered this trial, all of whom were considered evaluable for response and toxicity assessment. The demographic data, sites of metastatic tumour and prior therapies are listed in Table 1. The median age was 55 years (range 29–75), and the median WHO performance status was 1 (range 0–2). Except for 13 patients, all had multiple metastases involving two or more organ systems with predominant visceral, bone and soft-tissue sites in 39, six and eight patients respectively. Nineteen patients had received hormonal therapy for advanced disease, and palliative first-line chemotherapy was given to 16 women. Previous chemotherapy consisted of cyclophosphamide, methotrexate and 5-fluorouracil (CMF) in four patients and anthracycline-containing regimens in 12 patients. A total of 256 courses were administered to the 53 patients. The median number of treatment cycles was five (range 1–6) and the median duration of follow-up at the time of this analysis was 14 months (range 12–26).

### Response to therapy

Anti-tumour responses, according to the patients' pretreatment status, are shown in Table 2. The overall response rate was 47% for all 53 patients (95% confidence interval 33–61%), including five CRs (9%) and 20 PRs (38%). Nineteen patients (36%) showed stabilization of disease lasting more than 3 months, and in only nine patients (17%) was the disease progression not influenced by chemotherapy. The median time to response for all patients was 2 months (range 0.8–5.5). The median duration of response in all patients was 9.5 months (range 4.0–21) and the median time to treatment failure was 9.0 months, with a range of 2–23 months.

**Table 2** Objective response related to prior therapy

| | No prior chemotherapy ($n$ = 37) | Pretreated ($n$ = 16) |
|---|---|---|
| Complete remission | 5 (13%) | – |
| Partial remission | 17 (46%) | 3 (19%) |
| No change | 10 (27%) | 9 (56%) |
| Progression | 5 (14%) | 4 (25%) |
| Overall response rate | 22 (59%) | 3 (19%) |
| Median time to response (months) | 2.0 | 2.2 |
| Median response duration (months) | 9.5 | 10.6 |
| Median time to progression | 10.5 | 7.0 |
| Median survival (months) | > 13.0 | 11.0 |

Among the 37 chemotherapeutically naive patients with metastatic disease, five women (13%) achieved CR and 17 (46%) PR. The predominant site of tumour involvement in patients who experienced CR was visceral in three and soft tissue in two patients; apart from one patient, all had multiple metastases. Fifteen of 17 patients (88%) who achieved PR had multiple metastases with predominant visceral (65%) and soft tissue (35%) disease. The median duration of response in previously untreated patients was 9.5 months (range 4–21) and median time to progression was 10.5 months (range 2–23).

Among the 16 patients who had received prior palliative chemotherapy (including 12 patients who had received anthracyclines), three (19%) responded (three PR), nine had SD and tumours progressed in four. One patient with refractory disease who achieved PR had multiple skeletal lesions, while the other two patients had multiple lung and liver metastases; all had failed previous chemotherapy containing epirubicin and cyclophosphamide. Duration of response in chemotherapeutically pretreated patients was 6, 11.5 and 14.5 months and median time to progression was 7.0 months (range 2–19).

### Toxicity

All 53 patients who received a total of 256 cycles of therapy (512 administrations of vinorelbine), were assessable for toxicity. Side-effects associated with treatment are listed in Tables 3 and 4. The dose-limiting toxicity was myelosuppression. Leucopenia occurred in 40 patients (75%) and was grade 3 or 4 in 14 patients (26%). The median nadir WBC count was 3600 μl$^{-1}$ (range 250–38 720 μl$^{-1}$) and occurred at day 9 (as a median). The time to WBC count recovery to more than 3000 μl$^{-1}$ was short, i.e. 96% of episodes of leucopenia resolved within 7 days. The variations in granulocyte counts paralleled those of WBCs; the median nadir of granulocyte counts was 1850 μl$^{-1}$ (range 50–21 270 μl$^{-1}$). Thrombocytopenia was rather uncommon and rarely severe; it was noted in a total of ten patients (19%), three of whom had grade 3 (6%). There were no episodes of bleeding. The median nadir platelet count was 219 000 μl$^{-1}$ (range 37 000–637 000 μl$^{-1}$), with no evidence of a cumulative nature of this side-effect. Only four patients (8%) developed grade 3 anaemia requiring packed red blood cell (RBC)

© Cancer Research Campaign 1998

**Table 3** Highest grade of haematological toxicity experienced ($n = 53$)

| | Number of patients (%) with toxic effects according to WHO | | | |
|---|---|---|---|---|
| | Grade 1 | Grade 2 | Grade 3 | Grade 4 |
| Leucopenia | 10 (19%) | 16 (30%) | 11 (21%) | 3 (6%) |
| Neutropenia | 10 (19%) | 12 (23%) | 15 (28%) | 4 (8%) |
| Thrombocytopenia | 4 (8%) | 3 (6%) | 3 (6%) | – |
| Anaemia | 11 (21%) | 10 (19%) | 4 (8%) | – |

**Table 4** Highest grade of non-haematological toxicity experienced ($n = 53$)

| | Number of patients (%) with toxic effects according to WHO | | | |
|---|---|---|---|---|
| | Grade 1 | Grade 2 | Grade 3 | Grade 4 |
| Nausea/vomiting | 14 (26%) | 13 (25%) | 1 (2%) | – |
| Stomatitis | 12 (23%) | 7 (13%) | 3 (6%) | – |
| Diarrhoea | 9 (17%) | 5 (9%) | – | – |
| Alopecia | 6 (11%) | 3 (6%) | 1 (2%) | – |
| Infection | 8 (15%) | 4 (8%) | 1 (2%) | 1 (2%) |
| Phlebitis | 8 (15%) | 4 (8%) | – | – |
| Neurotoxicity | | | | |
| Peripheral | 6 (11%) | – | – | – |
| Constipation | 8 (15%) | 5 (9%) | – | – |
| Anorexia | 2 (4%) | – | – | – |

transfusion, whereas mild anaemia was recorded in 21 patients (40%). The median nadir of haemoglobin was 11.1 g $dl^{-1}$ (range 6.0–14.5 g $dl^{-1}$). Fourteen patients developed documented infection and two of them required hospitalization for sepsis.

Non-haematological side-effects are listed in Table 4. Gastrointestinal toxicity was the most frequently encountered non-haematological side effect (53%), although symptoms were generally mild, confined to the day of drug administration and responsive to standard antiemetic therapy. Stomatitis was seen in 22 patients (38%) and diarrhoea in 14 patients (26%). Chemically induced phlebitis was observed in 12 patients (23%) and six patients (12%) developed mild peripheral neurotoxicity. Constipation was noted in 13 patients (25%). Uncommon non-myelosuppressive toxicities included alopecia in ten (19%) and anorexia in two patients (4%). There was no G-CSF-related toxicity recorded in our trial.

Nine patients (17%) had at least one treatment delay of 1 week at some time during therapy and the total number of delayed courses was 13 (5%). The reasons for delayed courses were haematological in five, non-haematological in five and other reasons (Port-a-Cath implantation, urological surgery, orthopaedic surgery) in three.

Twelve patients (23%) had a 25% dose reduction of cytotoxic drugs during treatment according to the study protocol because of severe haematological ($n = 6$) or other systemic toxicities ($n = 4$) or both ($n = 2$). Dose intensity was calculated for each patient and for each drug. The mean given dose intensity of the combination was 96% of the projected dose with no significant difference between first-line (96%) and second-line patients (93.5%). The mean dose of vinorelbine was 19.1 mg $m^{-2}$ $week^{-1}$ (range 15.2–20 mg $m^{-2}$ $week^{-1}$) and the mean dose of 5-FU was 478 mg $m^{-2}$ $week^{-1}$ (range 396–500 mg $m^{-2}$ $week^{-1}$).

### Survival

As of December 1996, with a median follow-up duration of 14 months (range 12–26), 24 of all 53 patients entered have died because of progressive disease, except one, who expired as a consequence of coincident cardiovascular disease. Twenty-nine patients are still alive with metastatic disease, of whom 16 had received other oncological therapy (chemotherapy ± hormonotherapy) after subsequent PD. The median survival duration has not been reached yet and was > 13.0 months (range 1.5–26+) for previously untreated patients and 11.0 months (range 3.5–24+) in those who had received prior first-line chemotherapy.

### DISCUSSION

Most patients with metastatic breast cancer will receive systemic chemotherapy at some point during the course of their disease. In the United States and in Europe, the standard first-line chemotherapeutic regimen is usually either CMF (cyclophosphamide 600 mg $m^{-2}$ on days 1 and 8, methotrexate 40 mg $m^{-2}$ on days 1 and 8, 5-fluorouracil 600 mg $m^{-2}$ on days 1 and 8) or an anthracycline-containing combination such as cyclophosphamide/doxorubicin/5-fluorouracil (FAC). Several randomized trials show that response rates are consistently higher for anthracycline-containing regimens than those for CMF; however, time to disease progression and overall survival are only minimally prolonged, if at all, and the toxicity associated with the anthracylines is substantially higher (Hayes et al, 1987). Other palliative therapeutic strategies, including the use of alternating non-cross-resistant regimens, designs based on kinetic recruitment and, especially, administration of high doses of chemotherapy with or without autologous bone marrow or peripheral stem cell transplantation seem encouraging (Ayash et al, 1994; Hayes et al, 1995). These

© Cancer Research Campaign 1998

results, however, are commonly achieved at the expense of major toxic effects and warrant further confirmation, as they are necessarily associated with some degree of patient selection. For the large majority of patients presenting with metastatic breast cancer, a still incurable disease, research into new agents and novel combinations capable of achieving greater response rates with acceptable toxicity thus remains a priority.

Based on clinical trials of vinorelbine that have established the safety and efficacy of this vinca alkaloid as first- and second-line chemotherapy for the treatment of metastatic breast cancer (Cannobio et al, 1989; Garcia-Conde et al, 1992; Degardin et al, 1994; Fumoleau et al, 1994; Gasparini et al, 1994; Romero et al, 1994; Touissant et al, 1994; Weber et al, 1995), substantial activity for combination regimens including this agent would be anticipated. Among several such combination regimens that have already been investigated in patients with advanced breast cancer (Scheithauer et al, 1993; Spielmann et al, 1994; Fabi et al, 1995; Nole et al, 1995; Kornek et al, 1996), the combination of vinorelbine and doxorubicin has probably shown the most promising activity; there was a relatively high rate of cardiotoxicity (10%) however, resulting in three (4%) treatment-associated deaths (Spielmann et al, 1994), and a recently performed comparative study of VNB combined with doxorubicin vs doxorubicin alone failed to show any therapeutic difference between the two arms (Noris et al, 1996).

In the present study, we report the first results of front-line chemotherapy of advanced breast cancer with a combination of vinorelbine, 5-fluorouracil and l-leucovorin. The rationale for this combination was the documented activity of these agents in advanced breast cancer, their different, potentially additive or even synergistic mechanism of action and their non-overlapping toxicity profile except for myelosuppression, which might be overcome by prophylactic use of a supportive cytokine such as G-CSF. In an attempt to further alleviate dose-limiting neutropenia/other systemic toxicities and thus allow administration of full drug doses, we have also decided to use the laevorotatory isomer of LV (rather than racemic LV), as well as an extended administration schedule of 5-FU (2-h infusion rather than i.v. bolus injection; Machover et al, 1992; Valone, 1993; Scheithauer et al, 1997). Based on our previous experience with high-dose vinorelbine (Scheithauer et al, 1993), which was not associated with an increased rate of neurotoxicity, a 40 mg $m^{-2}$ dose of the semi-synthetic vinca alkaloid was given every 2 weeks.

Our results suggest an encouraging anti-tumour activity. Twenty-two of 37 (59%) patients with disseminated disease who had not received previous chemotherapy achieved objective remissions within a median time of only 8 weeks. It seems noteworthy that the therapeutic effectiveness was not influenced in these patients by the extent of disease (80% of responding patients had multiple tumour sites) or predominant visceral disease (64% of responses), i.e. factors that have previously been demonstrated to be related to an adverse prognosis and poor outcome (Falkson et al, 1991). The clinical responses achieved were durable and, after a follow-up duration of 14 months, median survival has not been reached yet. A lower response rate of only 19% was noted in our 16 patients failing previous palliative anthracycline or CMF-based chemotherapy; these therapeutic results, in fact, seem to overlap with those achievable with vinorelbine alone. This observation could be related, however, to the small pretreated study subpopulation, the somewhat lower dose intensity of cytotoxic drugs given to these patients when compared with first-line chemotherapy patients and/or an accumulation of adverse prognostic features.

Granulocytopenia was the most frequent and dose-limiting side-effect of this regimen, although it was generally mild to moderate, always rapidly reversible and rarely associated with infectious complications. Vinorelbine-associated neurological symptoms mainly manifested as paraesthesia, hypoaesthesia or autonomic neuropathy causing constipation. The overall incidence was comparable with single-agent or other combination regimens using conventional doses of vinorelbine (Hohnecker et al, 1994). All other non-haematological toxicities were of mild to moderate intensity and were recorded in only a minority of our patients.

In conclusion, the results of this study indicate that vinorelbine, 5-FU, LLV plus G-CSF is an effective first-line treatment for metastatic breast cancer. Its advantage over other commonly used cytotoxic combinations is its excellent tolerability, particularly the very low incidence of nausea and vomiting or alopecia. Based on its potentially favourable therapeutic index, a comparative trial with standard combinations such as CMF or FAC (5-fluorouracil 600 mg $m^{-2}$ on day 1, adriamycin 60 mg $m^{-2}$ on day 1, cyclophosphamide 600 mg $m^{-2}$ on day 1), including formal measurements of quality of life, seems warranted. The therapeutic potential of this combination regimen in pretreated patients, however, warrants further investigation.

### ACKNOWLEDGEMENT

This study was supported in part by the Austrian Cancer Society, Section of Niederösterreich.

### REFERENCES

Anderson JR, Bernstein L and Pike MC (1982) Approximate confidence intervals for probabilities of survival and quantiles in life-table analysis. *Biometrics* **38**: 407–416

Ayash LJ, Elias A, Wheeler C, Reich E, Schwartz G, Mazanet R, Tepler I, Warren D, Gonin R, Schnipper L, Frei E and Antman K (1994) Double dose-intensive chemotherapy with autologous marrow and peripheral-blood progenitor support for metastatic breast cancer: a feasibility study. *J Clin Oncol* **12**: 37–44

Binet S, Chaineau E, Fellous A, Cataste H, Krikorian A, Cozuzinier JP and Meininger V (1990) Immunofluorescence study of the action of navelbine, vincristine and vinblastine on mitotic axonal microtubules. *Int J Cancer* **46**: 262–266

Bonadonna G, Vallagussa P, Moliterni A, Zambanetti M and Brambilla C (1995) Adjuvant cyclophosphamide, methotrexate, and fluorouracil in node positive breast cancer: the results of 20 years follow-up. *New Engl J Med* **14**: 332–341

Cannobio L, Boccardo F, Pastorini G, Brema F, Martini C, Resasco M and Santi L (1989) Phase II study of navelbine in advanced breast cancer. *Semin Oncol* **16**: 33–36

Degardin M, Bonneterre J, Hecquet B, Pion JM, Adenis A, Horner D and Demaille A (1994) Vinorelbine (navelbine) as a salvage treatment for advanced breast cancer. *Ann Oncol* **5**: 424–426

Fabi A, Tonachella R, Savarese A, Cirulli S, Tomao S, Conte E and Cognetti F (1995) A phase II trial of vinorelbine and thiotepa in metastatic breast cancer. *Ann Oncol* **6**: 187–189

Falkson GF, Gelman R, Falkson CI, Glick J and Harris J (1991) Factors predicting for response, time to treatment failure, and survival in women with metastatic breast cancer treated with DAVTH: a prospective Eastern Cooperative Oncology Group study. *J Clin Oncol* **9**: 2153–2161

Fumoleau P, Delgado FM, Delozier T, Monnier A, Gil Delgado, MA, Kerbrat P, Garcia-Giralt, E, Keiling R, Namer M, Clason MT, Goudier MJ, Chollet P, Lecourt L and Mountouquet P (1994) Phase II trial of weekly intravenous vinorelbine in first-line advanced breast cancer chemotherapy. *J Clin Oncol* **11**: 1245–1252

Garcia-Conde J, Lluch A, Casado A, Gervasio H, de Oliveira C, de Pablo JL, Gorostiago J, Giron GC, Cervantes A, Martinez A, Pezous N, Delgado FM and

Diaz Rubio E (1992) Phase II trial with navelbine in advanced breast cancer. *Breast Cancer Res Treat* **23**: 143–145

Gasparini G, Caffo O, Barni S, Frontini L, Testolin A, Guglielmi RB and Ambrosini G (1994) Vinorelbine is an active antiproliferative agent in pretreated advanced breast cancer patients: a phase II study. *J Clin Oncol* **12**: 2094–2101

Hayes DF and Henderson IC (1987) CAF in metastatic breast cancer: standard therapy or another effective regimen. *J Clin Oncol* **5**: 1497–1499

Hayes DF, Henderson C and Schapiro CL (1995) Treatment of metastatic breast cancer: present and future aspects. *Semin Oncol* **22** (suppl. 5): 5–21

Hohnecker JA (1994) A summary of vinorelbine (navelbine) safety data from North American clinical trials. *Semin Oncol* **21** (suppl. 10): 42–47

Kornek GV, Haider K, Kwasny W, Hejna M, Raderer M, Meghadadi S, Burger D, Schneeweiss B, Depisch D and Scheithauer W (1996) Effective treatment of advanced breast cancer with vinorelbine 74, mitomycin C plus human granulocyte colony-stimulating factor. *Br J Cancer* **74**: 1668–1673

Lopirinzi CL (1989) 5-fluorouracil with leucovorin in breast cancer. *Cancer* **63**: 1045–1047

Machover D, Grison X, Goldschmidt E, Zittoun J, Lotz JP, Metzger G, Richaud J, Hannoun L, Marquet J, Guillot T, Salmon R, Sezeur A, Mauban S, Parc R and Izrael V (1992) Fluorouracil combined with the pure (6S)-stereoisomer of folinic acid in high doses for treatment of patients with advanced colorectal carcinoma: a phase I–II study. *J Natl Cancer Inst* **84**: 321–327

Margolin LA, Doroshow JH, Akman SA, Leong LA, Morgan RJ, Raschko JW, Somlo G and Blevins C (1992) Effective initial therapy of advanced breast cancer with fluorouracil and high-dose, continuous infusion calcium leucovorin. *J Clin Oncol* **10**: 1278–1283

Miller AB, Hoogstraten B and Staquet M (1981) Reporting results of cancer treatment. *Cancer* **147**: 207–214

Nole F, De Braud F, De Pas M, Castagna L, Covelli AL and Aapro S (1995) A phase I study of navelbine and fluorouracil plus folinic acid in patients with metastatic breast cancer (abstract 431). In *Proceedings of the 5th International Cancer Congress on Anticancer Chemotherapy, Paris, 31 Jan.–3 Feb.*

Noris B, Pritchard K and James K (1996) A phase III comparative study of vinorelbine (VNB) combined with doxorubicin (DOX) versus doxorubicin alone in metastatic/recurrent breast cancer (MBC): a National Cancer Institute of Canada (NCI CTG) study (abstract). *Proc ASCO* **15**: 98

Ribas A, Albanell J, Bellmunt, Soli-Calvo LA, Bermejo B, Gallardo E, Vidal R, Vera R, Eres N, Carulla J and Baselga J (1996) Five-day course of granulocyte colony-stimulating factor in patients with prolonged neutropenia after adjuvant chemotherapy for breast cancer is a safe and cost-effective schedule to maintain dose-intensity. *J Clin Oncol* **14**: 1573–1580

Romero A, Rabinovich MG, Vallejo CT, Perez JE, Rodriguez R, Cuevas MA, Machiavelli M, Lacava JA, Longhi M, Romero Acuna, L, Amato S, Barieri R, Sabatini C and Leone BA (1994) Vinorelbine as first-line chemotherapy for metastatic breast carcinoma. *J Clin Oncol* **12**: 336–341

Scheithauer W, Kornek GV, Haider K, Kwasny W, Raderer M, Tueni C, Koperna-Mach K and Depisch D (1993) Effective second-line chemotherapy for advanced breast cancer with navelbine and mitomycin C. *Breast Cancer Res Treat* **26**: 49–53

Scheithauer W, Kornek GV, Marczell A, Salem G, Karner J, Kovats E, Burger D, Greiner R, Pidlich J, Schneeweiss B, Raderer, M, Rosen H and Depisch D (1997) Fluorouracil plus racemic leucovorin versus fluorouracil combined with the pure l-isomer of leucovorin for the treatment of advanced colorectal cancer: a randomized phase III study. *J Clin Oncol* **15**: 908–914

Schüller J, Czejka M and Piertrzak C (1996) Serum and tissue levels of l-folinic acid (FA) after iv bolus of either racemic (d,l) FA or pure l-enantiomer (l-FA) (abstract). *Proc Am Soc Clin Oncol* **15**: 175

Spielmann M, Dorvalt T, Turpin F, Antoine E, Jouve M, Maylevin F, Lacombe D, Rousse J, Pouillart P, Tursz T and Merle S (1994) Phase II trial of vinorelbine/doxorubicin as first-line therapy of advanced breast cancer. *J Clin Oncol* **12**: 1764–1770

Swain SM, Lippman ME, Egan EF, Drake JC, Steinberg SM and Allegra CJ (1989) Fluorouracil and high-dose leucovorin in previously treated patients with metastatic breast cancer. *J Clin Oncol* **7**: 890–899

Toussaint C, Izzo J, Spielmann M, Merle S, May-Levin F, Armand JP, Lacombe D, Tursz, Sunderland M, Chabot GG and Cvitkovic E (1994) Phase I/II trial of continuous infusion vinorelbine for advanced breast cancer. *J Clin Oncol* **12**: 2102

Valagussa P, Bonadonna G, Veronesi A, Harvey H, Hutchins L, Bigley J and Hohneker J (1978) Patterns of relapse and survival following radical mastectomy. *Cancer* **41**: 1170–1172

Valone FH, Gandara DR, Luce JA, Wall S, Perez EA, Braham N, George M and Letvak L (1993) Phase I trial of 5-day-infusion of l-leucovorin plus daily bolus 5-fluorouracil in patients with advanced gastrointestinal malignancies. *Cancer Chemother Pharmacol* **32**: 215–220

Weber BL, Vogel C, Jones S, Harvey H, Hutchins L, Bigley J and Hohneker J (1995) Intravenous vinorelbine as first-line and second-line therapy in advanced breast cancer. *J Clin Oncol* **13**: 2722–2730

Zittoun J (1993) Pharmacokinetics and in vitro studies of l-leucovorin. Comparison with the d- and d,l-leucovorin. *Ann Oncol* **4** (suppl. 2): 1–5

© Cancer Research Campaign 1998